# STAT3 modulates cigarette smoke-induced inflammation and protease expression

**DOI:** 10.3389/fphys.2013.00267

**Published:** 2013-10-01

**Authors:** Patrick Geraghty, Anne E. Wyman, Itsaso Garcia-Arcos, Abdoulaye J. Dabo, Sonya Gadhvi, Robert Foronjy

**Affiliations:** ^1^Division of Pulmonary and Critical Care Medicine, St. Luke's Roosevelt Health Sciences Center, New YorkNY, USA; ^2^Department of Medicine, Columbia University College of Physicians and Surgeons, New YorkNY, USA

**Keywords:** inflammation, cytokines, proteases, apoptosis, signaling, lung, COPD

## Abstract

Signal transducer and activator of transcription-3 (STAT3) regulates inflammation, apoptosis, and protease expression, which are critical processes associated with airway injury and lung tissue destruction. However, the precise role of STAT3 in the development of airway diseases such as chronic obstructive pulmonary disease (COPD) has not been established. This study shows that cigarette smoke activates STAT3 in the lungs of mice. Since cigarette smoke activated STAT3 in the lung, we then evaluated how the loss of STAT3 would impact on smoke-mediated lung inflammation, protease expression, and apoptosis. STAT3^+/+^ and STAT3^−/−^ mice were exposed to 8 days of cigarette smoke. Compared to the STAT3^+/+^ mice bronchoalveolar lavage fluid (BALF) cellularity was significantly elevated in the STAT3^−/−^ mice both before and after cigarette smoke exposure, with the increase in cells primarily macrophages. In addition, smoke exposure induced significantly higher BALF protein levels of Interleukin-1α (IL-1α), and monocyte chemotactic protein-1 (MCP-1) and higher tissue expression of keratinocyte chemoattractant (KC) in the STAT3^−/−^ mice. Lung mRNA expression of MMP-12 was increased in STAT3^−/−^ at baseline. However, the smoke-induced increase in MMP-10 expression seen in the STAT3^+/+^ mice was not observed in the STAT3^−/−^ mice. Moreover, lung protein levels of the anti-inflammatory proteins SOCS3 and IL-10 were markedly lower in the STAT3^−/−^ mice compared to the STAT3^+/+^ mice. Lastly, apoptosis, as determined by caspase 3/7 activity assay, was increased in the STAT3^−/−^ at baseline to levels comparable to those observed in the smoke-exposed STAT3^+/+^ mice. Together, these results indicate that the smoke-mediated induction of lung STAT3 activity may play a critical role in maintaining normal lung homeostasis and function.

## Introduction

Chronic obstructive pulmonary disease (COPD) is now the third leading cause of death in the United States (Murphy et al., 2012) and it is projected to become the third leading cause worldwide within the next 20 years (Raherison and Girodet, 2009). In contrast to other diseases, the age-adjusted mortality for COPD has increased over the past 30 years (Miller et al., 2000). Current pharmacological therapies improve lung function and slow disease progression but they have not been shown to impact on COPD mortality (Kim and Criner, 2013). Cigarette smoke is the major etiologic factor associated with this disorder and prolonged exposure to cigarette smoke induces damaging inflammatory, apoptotic, and proteolytic responses in the lung (Macnee, 2007). These biological processes lead to dysfunctional matrix remodeling that cause airway obstruction and lung tissue destruction (Cornwell et al., 2010). A better understanding of the varied signaling processes that mediate the loss of normal airway function is needed to more effectively address the underlying mechanisms of this disease.

Signal transducer and activator of transcription 3 (STAT3) is a transcription factor that mediates IL-6 signaling responses. Upon binding to the gp130 receptor, IL-6 activates Janus kinases (JAKs), which phosphorylate STAT3 thereby inducing its nuclear translocation and transcriptional activation. In addition to IL-6 signaling, STAT3 can be activated by EGFR, PDGFR, and Src kinase. Once activated, STAT3 binds the enhancer element in the promoter region of acute-phase genes, known as the acute-phase response element (Gerhartz et al., 1996) and induces the expression of numerous pro-inflammatory genes in the lung (Saleh et al., 2009). STAT3 was recently shown to be required for the development of allergic inflammation in a mouse model of asthma (Simeone-Penney et al., 2007). Indeed, the loss of STAT3 expression within the airway epithelium decreased the expression of Th2 cytokines, lowered airway inflammation and prevented the development of airway hyperreactivity in an asthma model (Simeone-Penney et al., 2007). Conversely, transgenic expression of STAT3 within the alveolar epithelium of mice markedly induced cytokine expression and lung inflammation (Gobburu et al., 1998). Given these findings, STAT3 activation is believed to be a central factor in the induction of airway inflammatory responses. Nevertheless, the role of STAT3 in airway diseases remains to be determined.

Though STAT3 affects key disease processes, data on the effect of STAT3 in COPD is lacking. Upregulation of STAT3 and induction of genes associated with STAT3 expression have been documented in lung tissue samples from COPD patients (Qu et al., 2009). Since STAT3 expression is increased in COPD, our study examined how cigarette smoke exposure impacted on STAT3 activation in the lungs of mice over the course of 1 year. In addition, we utilized the cigarette smoke exposure model to determine how STAT3 directly affected key biological processes implicated in the development of COPD. To examine this, STAT3^+/+^ and STAT3^−/−^ mice were exposed to cigarette smoke in order to assess how the loss of STAT3 impacted on lung inflammation, apoptosis and protease expression.

## Methods

### Animal models

Three-month old 129X1-*Stat*^3*tm*1*Desi*^/J (STAT3^−/−^) and 129X1/SvJ controls (STAT3^+/+^) (Jackson Labs, Bar Harbor, ME) were used for these studies. The 129X1-*Stat*^3*tm*1*Desi*^/J mice have a mutant β isoform of STAT3 and only express the α isoform. C57Bl/6J mice (Jackson Labs) were used to kinetically examine the effects of cigarette exposure (from 1 day to 1 year of smoke exposure) on STAT3 activity in lung protein. All animal experiments were performed with approval from St. Luke's Roosevelt's Hospital Center's Institutional Animal Care and Use Committee approval.

### Cigarette smoke exposure protocol

In accordance with our previously published protocol (Geraghty et al., 2013), mice were exposed to cigarette smoke from 3R4F research cigarettes (University of Kentucky, Lexington, KY) in a specially designed whole body smoking apparatus (Teague Enterprises, Davis, CA) for 4 h a day at a total particulate matter concentration of 80 mg/m^3^. The mice had access to food and water and were able to move about freely during the period of smoke exposure. The mice were euthanized and bronchoalveolar lavage fluid (BALF) and lung tissue was collected 12 h following the last cigarette smoke exposure.

### qPCR

mRNA was isolated from lung tissue of room air and 8-day smoke exposed STAT3^+/+^ and STAT3^−/−^ mice using Trizol reagent (Life Technologies, Grand Island, NY). cDNA was prepared from this mRNA using Superscript (Life Technologies). Lung expression of Cathepsin S, Cathepsin K, matrix metalloproteinase-3 (MMP-3), MMP-7, MMP-8, MMP-9, MMP-10, MMP-12, MMP-13, tumor necrosis factor-α (TNF-α), IL-1β, IL-17, CD68, and KC was assessed by quantitative PCR using Taqman specific probes (Applied Biosystems, Grand Island, NY). Analyzed changes in gene expression in samples were related to another reference sample, usually room air treated STAT3^+/+^ mice where the reference sample is set to 1.

### Protease and cytokine measurements

IL-1α, IL-6, IL-10, IL-13, IL-17, IFN-γ, MCP-1, RANTES (**R**egulated on **A**ctivation, **N**ormal **T** cell **E**xpressed and **S**ecreted), and TNF-α were measured in BALF using a beads assay on the BioRad Bio-Plex 200 system (BioRad, Hercules, CA).

### Intracellular signaling

Lung tissue was subfractionated into cytosolic and nuclear fractions using a commercially available protein compartment kit (Millipore, Billerica, MA). Immunoblots were conducted on the cytosolic protein to assess levels of phospho-B cell lymphoma-2 protein (p-Bcl-2), Bcl-2, suppressor of cytokine signaling3 (SOCS3) and actin (all antibodies from Cell Signaling, Danvers, MA). The proteins were separated by sodium dodecyl sulfate polyacrylamide gel electrophoresis and then transferred to a nitrocellulose membrane. After blocking for 1 h with 5% milk protein (BioRad, Hercules, CA), the membranes were incubated for 1 h at room temperature with a 1:1000 dilution of primary antibody (p-Bcl2ser70 #2827, Bcl2 #2876, SOCS3 #2932, Actin #4967) in 2.5% bovine serum albumin (BSA). After washing, the membranes were then incubated with a 1:4000 dilution of horseradish peroxidase linked secondary antibody (#7074) in 2.5% BSA for 1 h. After washing, the membranes were incubated with Supersignal West Pico Luminol Enhancer Solution (Thermo Scientific, Rockford, IL) and then imaged on a ChemiDoc XRS Imaging System (BioRad).

### STAT3 activity

Lung nuclear protein from the lung tissue homogenates of C57Bl/6J mice exposed to 1 day, 8 days, 7 weeks or 1 year of cigarette smoke was used to determine STAT3 activity by conducting an oligonucleotide-binding assay (Active Motif, Carlsbad, CA). Of note, the lung tissue samples had undergone lung lavage prior to measuring STAT3 activity. Results are presented as relative activity compared to controls (%), which is the reference sample (usually room air treated STAT3^+/+^ mice).

### Caspase 3/7 activity assay

Caspase 3/7 activity was measured in the lung tissue whole lysates of control and smoke-exposed STAT3^+/+^ and STAT3^−/−^ mice using the Caspase-Glo 3/7 Assay System (Promega, Fitchburg, WI). Data is reported as relative luminescence units (RLU). RLU is determined by analysis of signal relative to background, using a Tecan Genios microplate reader.

### Statistical analyses

Data are expressed as means ± S.E.M. We determined statistical significance by one-way analysis of variance for multiple group analysis using GraphPad Prism Software (GraphPad, La Jolla, CA). Student *t*-tests (two tailed) were used throughout the study. All data sets are represented as range and mean ± standard error.

## Results

### Cigarette smoke induces STAT3 activation in mouse lungs

**Figure 1:**
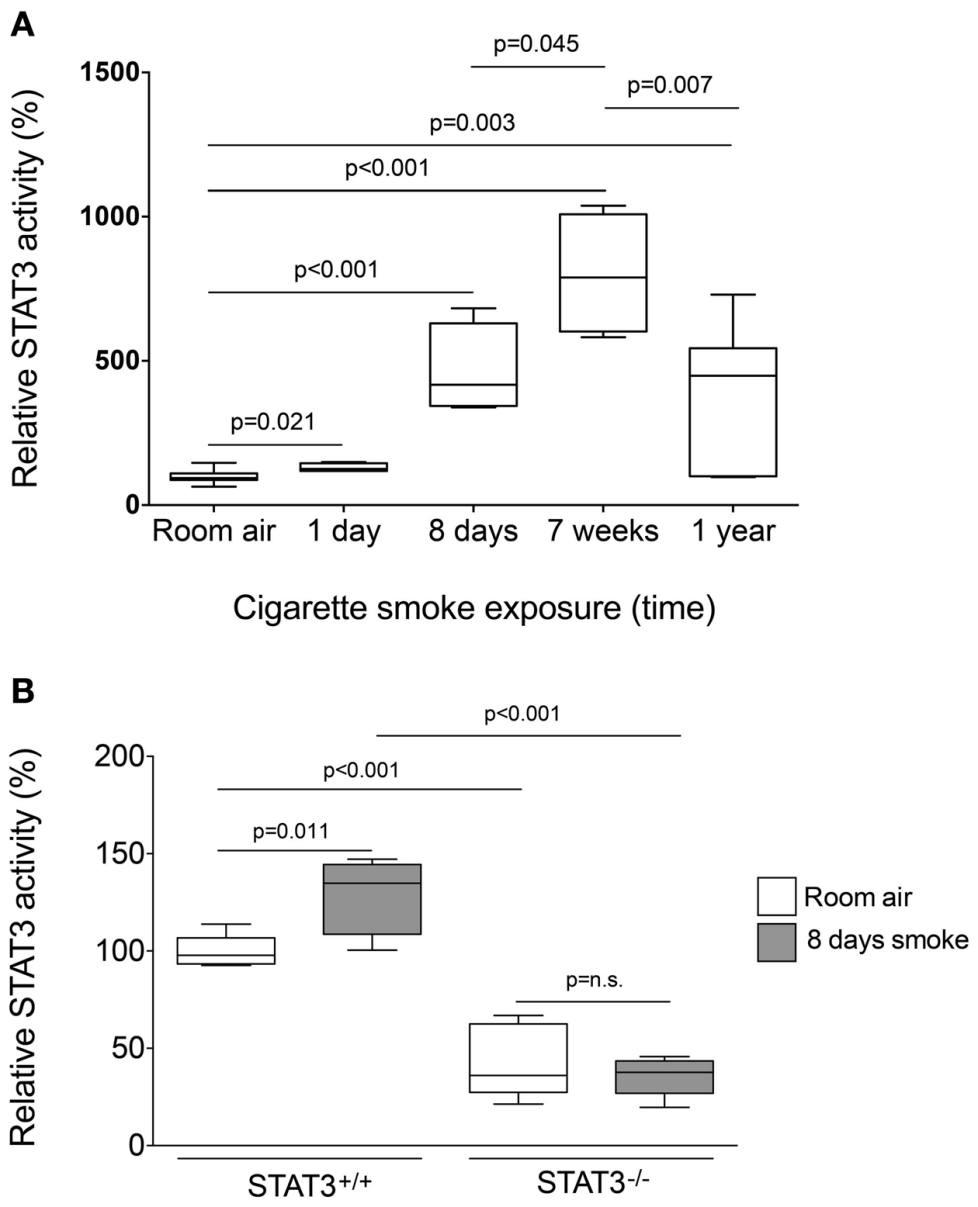
**Cigarette smoke exposure induces STAT3 activation in the lungs of exposed mice. (A)** Three-month old C57Bl/6J mice were exposed to cigarette smoke for 1 day, 8 days, 7 weeks, and 1 year. Nuclear protein fractions were prepared from the lung tissue and STAT3 activity was measured using a specific oligonucleotide-binding assay. **(B)** Lung nuclear proteins were examined for STAT3 activity from 129X1-*Stat*^3*tm*1*Desi*^/J (STAT3^−/−^) and 129X1/SvJ controls (STAT3^+/+^) mice after 8 days of either room air or cigarette smoke exposure. Data are reported as STAT3 activity relative to air-exposed STAT3^+/+^ mice. Plots show range and average ± S.E.M. *N* = 10 in each group. *p* values shown, comparing both treatments connected by a line. n.s. denotes no significant difference between groups.

To determine the STAT3 response to cigarette smoke, STAT3 activity was measured in the nuclear fraction of the lungs of C57Bl/6 mice subjected to varying periods of smoke exposure. A 30% increase in STAT3 activation was observed as early as one-day post cigarette smoke exposure (Figure 1). Moreover, 8 days exposure to cigarette smoke further activated STAT3 ~5 fold. Maximal activation occurred after 7 weeks though increased activity was observed up to 1 year of smoke exposure (Figure 1A). It is conceivable that down regulation of IL-6 receptors or the induction of protein inhibitor of activated STAT3 (PIAS3) may have diminished the smoke-mediated induction of STAT3 at the 1-year time point. However, this will need to be explored in future studies. Together, these results show that cigarette smoke causes a rapid and persistent activation of STAT3 in the lung.

### Increased inflammatory cells in the airways of STAT3^−/−^ mice

**Figure 2:**
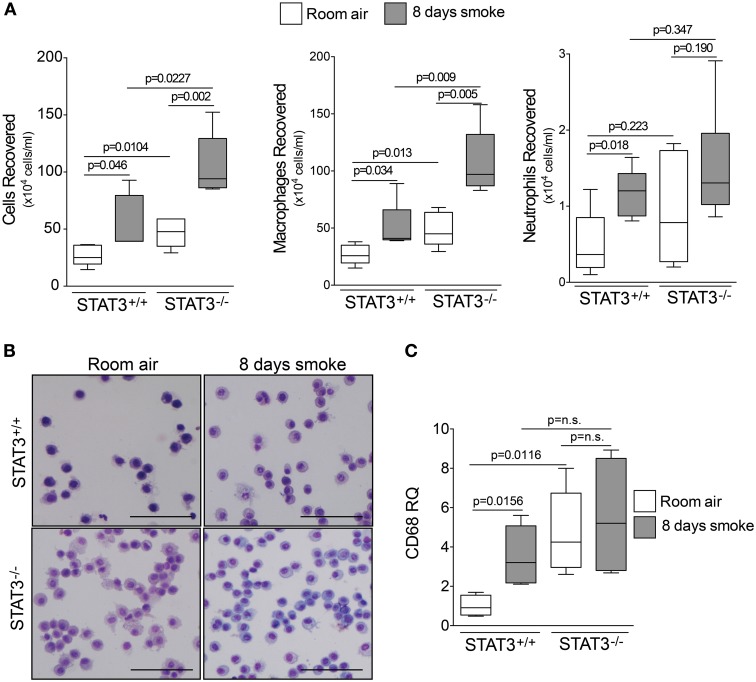
**STAT3 expression subdues BALF cellularity.** Three-month old STAT3^+/+^ and STAT3^−/−^ mice were exposed to room air (white bars) or cigarette smoke (black bars) for 4 h daily for 8 days. The mice were euthanized 12 h post the last smoke exposure and **(A)** BALF cellularity was measured by bright field microscopy. BALF macrophages and neutrophils were determined by quick diff staining. **(B)** A typical representative staining is shown here (Scale bar = 20 μM). **(C)** qPCR for macrophage marker CD68 also demonstrates higher levels of macrophages within the lung tissue. Data are reported as BALF cells (x 10,000). Plots show range and average ± S.E.M. *N* = 10 in each group. *p* values shown, comparing both treatments connected by a line. n.s. denotes no significant difference between groups.

Since cigarette smoke potently activated STAT3 in the lungs of mice, we aimed to determine how the loss of STAT3 would impact on smoke-mediated inflammatory responses. To address this question, we exposed 129X1-*Stat*^3*tm*1*Desi*^/J (STAT3^−/−^) and 129X1/SvJ (STAT3^+/+^) mice to 8 days of cigarette smoke exposure. Cigarette smoke increased STAT3 activity levels with the lung of the STAT3^+/+^ after 8 days of smoke exposure (Figure 1B). Cigarette smoke significantly increased BALF cellularity in the lungs of the STAT3^+/+^ mice (26.8 ± 3.7 × 10^4^ vs. 53.9 ± 13.3 × 10^4^ cells for room air and smoke exposed, respectively; *p* < 0.05) (Figure 2A). In comparison, the STAT3^−/−^ mice under room air conditions had significantly higher lung BALF cellularity than STAT3^+/+^ mice (26.8 ± 3.7 × 10^4^ vs. 48.2 ± 5.8 × 10^4^ cells for STAT3^+/+^ and STAT3^−/−^, respectively; *p* < 0.05). In fact, the BALF cellularity of room air exposed STAT3^−/−^ mice was comparable to that of STAT3^+/+^ mice that had been exposed to cigarette smoke for 8 days (Figure 2A). The increase in BALF cells was primarily macrophages (Figure 2B). Consistent with the increase in BALF cells that was measured in the BALF, we found significantly higher expression of the macrophage marker CD68 in the STAT3^−/−^ mice under room air conditions compared to the room air exposed STAT3^+/+^ mice (Figure 2C). Cigarette smoke exposure caused a two-fold increase in BALF cellularity in the STAT3^−/−^ mice compared to room air exposed STAT3^−/−^ mice (Figure 2A). This smoke-mediated increase was proportional to that experienced by the smoke-exposed STAT3^+/+^ mice though the STAT3^+/+^ had significantly lower levels of inflammatory cells post exposure compared to the STAT3^−/−^ mice. Together, these findings show that the lack of STAT3 activation alters immune cell infiltration in the lung.

### STAT3 expression impacts on lung cytokine and protease induction

**Figure 3:**
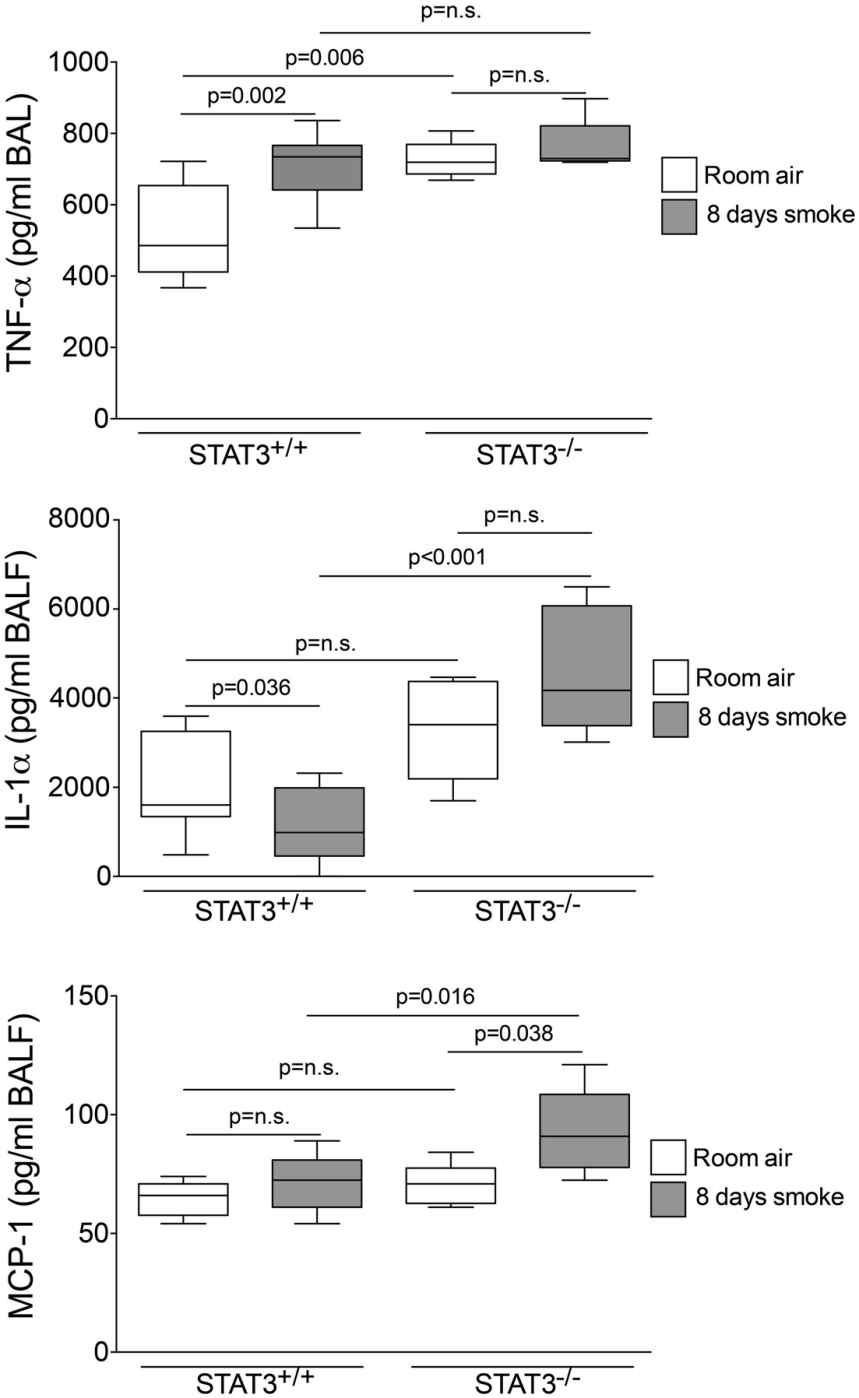
**STAT3 expression suppresses TNF-α, IL-1α, and MCP-1 BALF protein levels.** Bioplex assays measured BALF cytokines in room air and 8-day smoke-exposed STAT3^+/+^ and STAT3^−/−^ mice. Data are reported as cytokine level (pg/ml). Plots show range and average ± S.E.M. *N* = 10 in each group. *p* values shown, comparing both treatments connected by a line. n.s. denotes no significant difference between groups.

**Figure 4:**
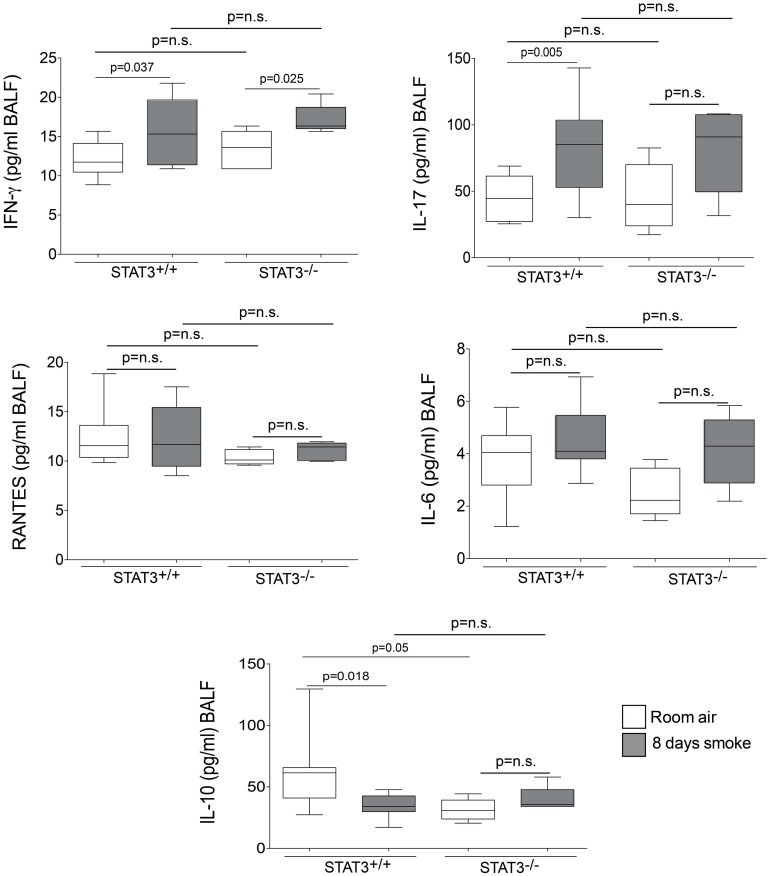
**STAT3 expression impacts on IL-10 BALF protein levels.** Bioplex assays measured BALF cytokines (IFN-γ, IL-17, RANTES, IL-6, and IL-10) in room air and 8-day smoke-exposed STAT3^+/+^ and STAT3^−/−^ mice. Data are reported as cytokine level (pg/ml). Plots show range and average ± S.E.M. *N* = 10 in each group. *p* values shown, comparing both treatments connected by a line. n.s. denotes no significant difference between groups.

**Figure 5:**
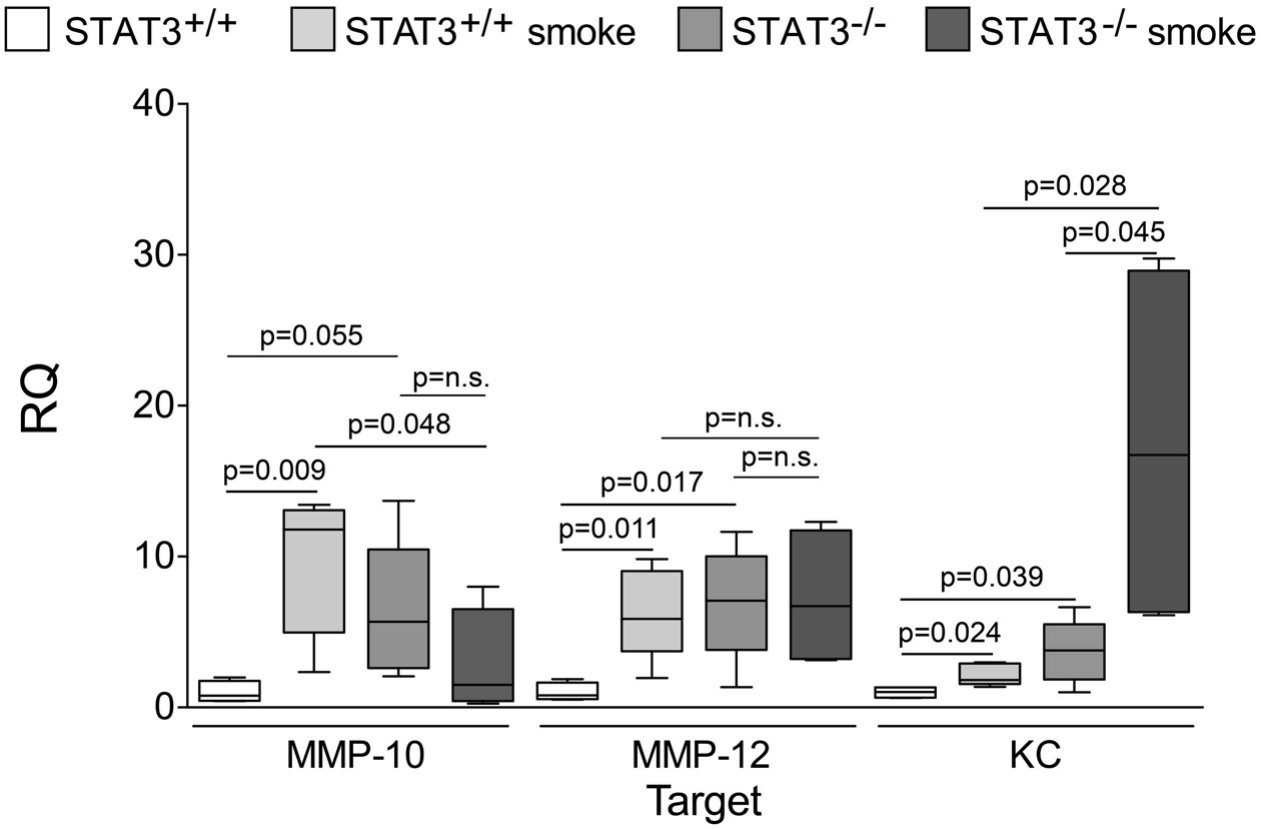
**Loss of lung STAT3 activity enhances gene expression of MMP-10 and MMP-12.** Quantitative PCR for mouse KC, MMP-10, and MMP-12 was conducted on mRNA isolated from the lung tissue of room air and 8-day smoke exposed STAT3^+/+^ and STAT3^−/−^ mice using specific Taqman probes. Data are reported as relative expression (RQ) to STAT3^+/+^ in room air conditions. Plots show range and average ± S.E.M. *N* = 10 in each group. *p* values shown, comparing both treatments connected by a line. n.s. denotes no significant difference between groups.

**Figure 6:**
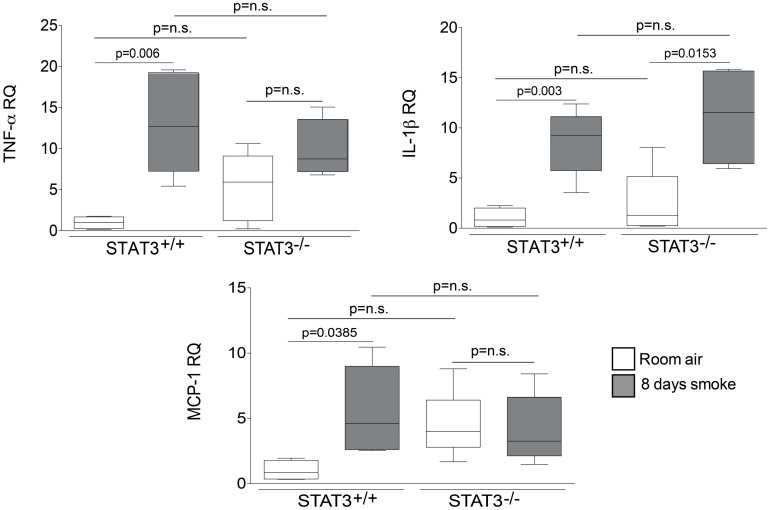
**STAT3 does not alter lung expression of TNF-α, IL-1β or MCP-1.** Quantitative PCR for mouse TNF-α, IL-1β or MCP-1 was conducted on mRNA isolated from the lung tissue of room air and 8-day smoke exposed STAT3^+/+^ and STAT3^−/−^ mice using specific Taqman probes. Data are reported as relative expression (RQ) to STAT3^+/+^ in room air conditions. Plots show range and average ± S.E.M. *N* = 10 in each group. *p* values shown, comparing both treatments connected by a line. n.s. denotes no significant difference between groups.

Cigarette smoke induces the expression of pro inflammatory cytokines and destructive proteases in the lung (Foronjy and D'Armiento, 2001; Wright et al., 2002). For this reason, we evaluated how the loss of STAT3 altered lung cytokine and protease expression in smoke-exposed mice. Under room air exposure conditions, TNF-α BALF protein levels were significantly higher in STAT3^−/−^ mice than in STAT3^+/+^ mice (~2-fold, *p* < 0.05) (Figure 3). In response to cigarette smoke, IL-1α BALF protein levels decreased in the STAT3^+/+^ mice (*p* < 0.05), but trended higher in the STAT3^−/−^ mice (Figure 3). In contrast, cigarette smoke increased MCP-1 BALF protein levels in the STAT3^−/−^ mice, but not in the STAT3^+/+^ mice (Figure 3). STAT3 had no effect on the smoke-mediated increase in IFN-γ and IL-17 BALF protein expression and neither cigarette smoke nor STAT3 expression altered RANTES or IL-6 BALF protein levels (Figure 4). Of note, IL-10 has potent anti-inflammatory effects in the lung (Mays et al., 2013) and BALF protein levels of the anti-inflammatory cytokine IL-10 were significantly lower in the STAT3^−/−^ mice under room air conditions (Figure 4). We next conducted quantitative PCR analyses to determine how STAT3 impacted on cytokine and protease expression within the lung tissue of the mice. We found that KC was regulated both by cigarette smoke exposure and STAT3 expression. Compared to STAT3^+/+^ mice, the STAT3^−/−^ mice had a ~3-fold increase in lung KC expression under room air conditions (Figure 5). Furthermore, cigarette smoke induced a 2-fold increase in KC expression in STAT3^+/+^ mice (*p* < 0.05), but a ~15-fold increase in STAT3^−/−^ mice (*p* < 0.05 vs. room air and vs. smoked wild type; Figure 5). In terms of proteases, cigarette smoke induced a ~10-fold increase in MMP-10 lung mRNA expression (*p* < 0.01) in the STAT3^+/+^ mice but not in the smoke-exposed STAT3^−/−^ mice (Figure 5). Importantly, lung MMP-12 mRNA expression was upregulated ~8-fold in the STAT3^−/−^ mice under room air conditions (*p* < 0.02). However, MMP-12 expression was comparable in STAT3^+/+^ and STAT3^−/−^ mice following cigarette smoke exposure. Similarly, TNF-α, IL-1β, and MCP-1 lung tissue expression were comparable in the STAT3^+/+^ and STAT3^−/−^ mice under smoke exposure conditions (Figure 6).

### Loss of STAT3 impacts on SOCS3 protein levels in the lung

**Figure 7:**
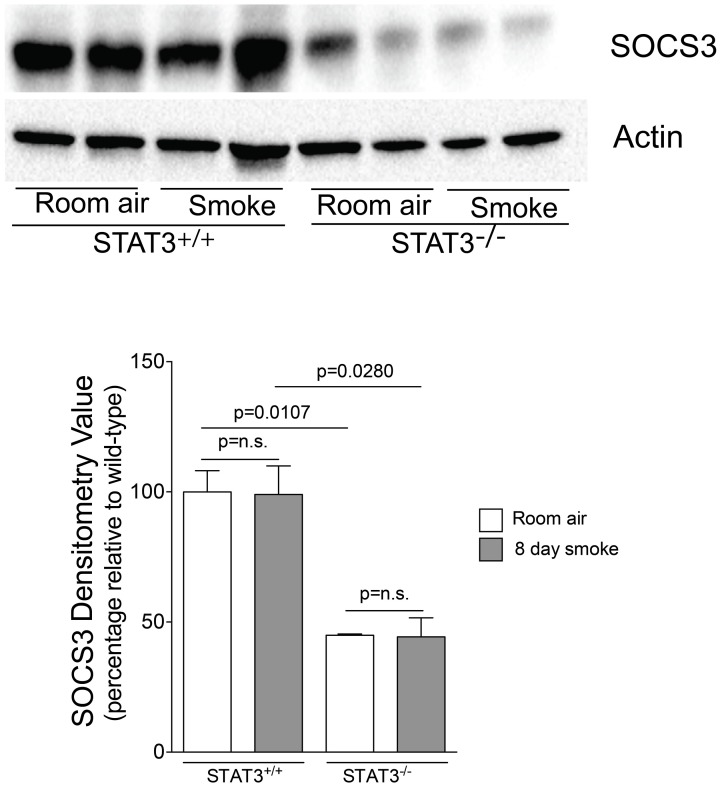
**STAT3 activity enhances lung SOCS-3 levels.** Lungs from STAT3^+/+^ and STAT3^−/−^ mice exposed to room air or smoke for 8 days were homogenized and SOCS3 and actin were detected by immunoblot. Densitometry analysis was performed and results are represented as SOCS3 protein relative to air-exposed STAT3^+/+^ mice. Actin levels were also used to normalize protein loading and are reflected in graph shown here.

Suppressor of cytokine synthesis 3 (SOCS3) counters inflammation by blocking JAK and their associated receptors to limit the duration and intensity of cytokine signaling (Babon and Nicola, 2012). Compared to STAT3^+/+^ mice, both SOCS3 protein expression was markedly lower in the STAT3^−/−^ mice (Figure 7). Thus, the loss of STAT3 expression may enhance lung inflammation by diminishing the anti-inflammatory responses mediated by SOCS3.

### Caspase 3/7 activity is increased in the lungs of the STAT3^−/−^ mice

**Figure 8:**
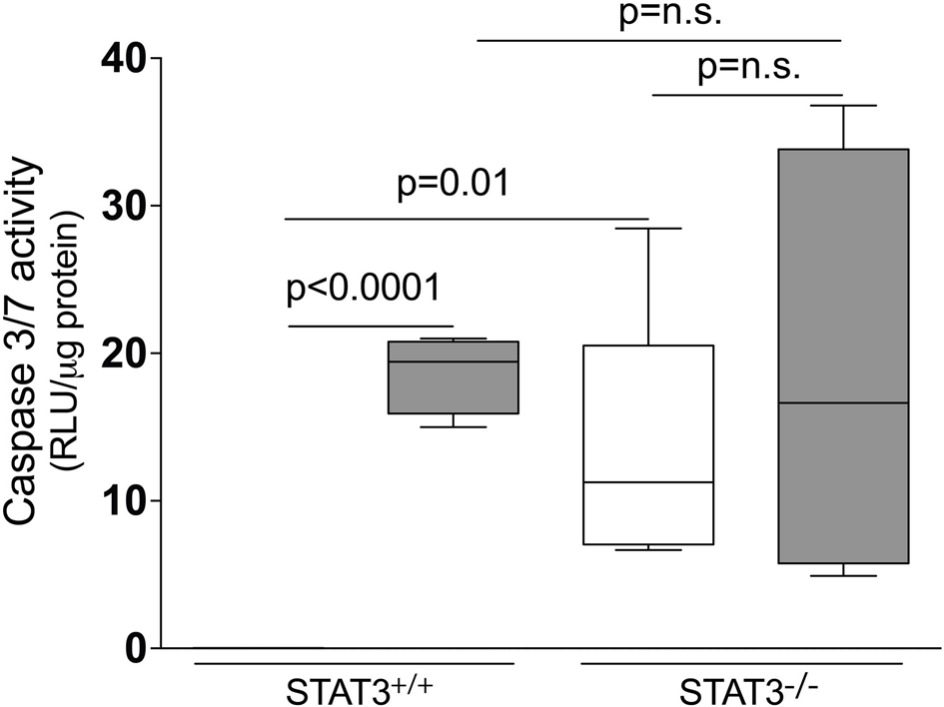
**Loss of STAT3 expression increases lung Caspase 3/7 activity.** Caspase 3/7 activity was measured from the lung tissue homogenates of control and 8-day smoke exposed STAT3^+/+^ and STAT3^−/−^ mice using a specific luminescence assay (Promega). Data are reported as relative luminescence units (RLU). Plots show range and average ± S.E.M. *N* = 10 in each group. *p* values shown, comparing both treatments connected by a line. n.s. denotes no significant difference between groups.

**Figure 9:**
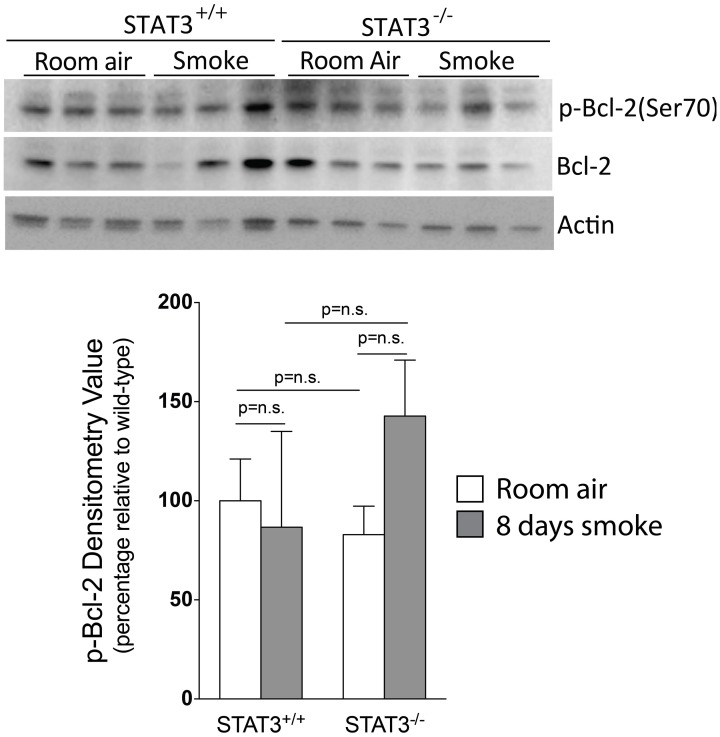
**Loss of STAT3 gives a trend increase in smoke induced Bcl-2 phosphorylation in mouse lungs.** Lungs from STAT3^+/+^ and STAT3^−/−^ mice exposed to room air or smoke for 8 days were homogenized and p-Bcl-2, Bcl-2, and actin were detected by immunoblot. Each group had replicates of 3. Densitometry analysis was performed and results are represented as p-Bcl-2 protein relative to air-exposed STAT3^+/+^ mice. Bcl-2 levels were also used to normalize protein loading and are reflected in graph shown here.

Apoptosis is a critical process in the development of emphysema (Tuder et al., 2003) and STAT3 regulates apoptotic responses *in vivo* (Chapman et al., 2000; Abell et al., 2005). Thus, we sought to determine how the loss of STAT3 expression impacted on apoptotic responses in the lungs of smoke-exposed mice. We measured caspase 3/7 activity, an important marker of apoptosis (Albee et al., 2007), in the lungs of room air and 8-day smoke-exposed STAT3^+/+^ and STAT3^−/−^ mice. Cigarette smoke induced a ~20 fold increase in caspase 3/7 activity in the smoke-exposed STAT3^+/+^ mice (*p* < 0.001) (Figure 8). Compared to the STAT3^+/+^ mice, the STAT3^−/−^ had a ~10 fold increase in caspase 3/7 activity at baseline (*p* < 0.01). However, the heightened activity did not increase further increase with cigarette smoke exposure in STAT3^−/−^ mice (Figure 8). STAT3 can regulate apoptosis by inducing the expression of the apoptotic inhibitor Bcl-2 (Gao and Ward, 2007). Given this, immunoblots for p-Bcl-2 and total Bcl-2 were conducted on lung tissue lysates from control and 8-day smoke-exposed STAT3^+/+^ and STAT3^−/−^ mice. The expression of both p-Bcl-2 and total Bcl-2 showed a trend toward an increase in STAT3^+/+^ mice exposed to smoke, while the trend was the opposite in the STAT3^−/−^ mice (Figure 9). However, the difference in p-Bcl-2 levels in the smoke exposed STAT3^+/+^ and STAT3^−/−^ mice did not reach statistical significance.

## Discussion

The role of STAT3 in inflammation is not clearly defined, with evidence supporting STAT3 activation having pro-inflammatory and anti-inflammatory roles (El Kasmi et al., 2006; Ruwanpura et al., 2012). These varied effects may be due to disease- and cell-specific mechanisms that determine the ultimate effect that STAT3 exerts in a biological system. The expression of STAT3 and its downstream-related genes is significantly increased in the lung tissue of COPD patients (Qu et al., 2009). This is significant as STAT3 is known to regulate inflammation, protease expression, and apoptosis (Li et al., 2011; Camporeale and Poli, 2012; Du et al., 2012), which are key biological processes in the pathogenesis of COPD (Chung and Adcock, 2008). For this reason, we sought to determine how the loss of STAT3 would impact on these important disease parameters in the cigarette smoke exposure model. This work shows for the first time that STAT3^−/−^ mice have enhanced inflammatory, proteolytic and apoptotic responses in response to cigarette smoke. Furthermore, the loss of STAT3 expression was associated with deficient anti-inflammatory responses in these mice. STAT3 has been viewed as a potential therapeutic target for the treatment of COPD (Gao and Ward, 2007). However, these findings suggest that STAT3 may have protective functions in the lung and inhibiting it could exacerbate the underlying mechanisms involved in cigarette smoke-induced lung diseases.

One of the most remarkable findings in the STAT3^−/−^ mice was the increased numbers of BALF cells, mostly macrophages, under room air or smoke exposure conditions. Macrophages are critical in COPD as these cells release proteases that induce dysfunctional airway remodeling (Shapiro et al., 1991; Shapiro, 1999, 2003). This enhanced macrophage response may have been due to the increased MCP-1 expression that was detected in the STAT3^−/−^ mice. MCP-1 is upregulated in COPD patients (Bracke et al., 2007) and induces mucin production and lung inflammation (de Boer et al., 2000; Monzon et al., 2011) characterized by the influx of macrophages (Hautamaki et al., 1997). It is somewhat surprising that the absence of STAT3 increased MCP-1 levels as several studies demonstrated that MCP-1 expression is positively regulated by STAT3 (Burysek et al., 2002; Chatterjee et al., 2009; Zhou et al., 2012). However, the effect of STAT3 is likely influenced by the cell type and physiologic context. Indeed, the loss of STAT3 expression in macrophages enhanced inflammatory responses and increased MCP-1 expression in thioglycollate-challenged mice (Matsukawa et al., 2005).

In addition to the changes in MCP-1 expression, baseline TNF-α levels were significantly increased in the STAT3^−/−^ mice. TNF-α, which is expressed by macrophages and other resident lung cells, induces intracellular signaling events that promote the development of emphysema (Riches et al., 1996; Churg et al., 2004). Thus, our findings indicate that STAT3 expression can impact on the disease by countering TNF-α expression in the lung. Of note, STAT3 regulated BALF TNF-α protein levels but not lung tissue TNF-α expression. This may indicate that STAT3 has a greater role on macrophage TNF-α expression than it does on lung tissue TNF-α expression. In support of this, prior studies have shown that the induction of SOCS3 by STAT3 counters TNF-α expression in macrophages (Berlato et al., 2002; Williams et al., 2004). However, further studies will be needed to definitively evaluate the effect of STAT3 within specific cell types in this model.

In addition to TNF-α, STAT3 also deterred the smoke-mediated induction of IL-1α in mice. This is significant as IL-1α drives the influx of neutrophils in the lungs of COPD patients (Botelho et al., 2011) and neutralizing IL-1α attenuates cigarette smoke-induced inflammation in mice (Pauwels et al., 2011). The enhanced inflammatory response in the STAT3^−/−^ mice was associated with increased MMP-12 expression. The fact that STAT3 negatively regulated MMP-12 is significant as this protease is upregulated in COPD patients (Demedts et al., 2006; Ilumets et al., 2007) and plays a critical role in smoke-induced emphysema in mice (Hautamaki et al., 1997). While the loss of STAT3 increased MMP-12 expression, STAT3^−/−^ mice had significantly lower lung expression of MMP-10 following cigarette smoke exposure. This is important as MMP-10 was found to decrease inflammation in a mouse model of experimental colitis (Koller et al., 2012). Thus, it is conceivable that the loss of STAT3 expression can enhance lung inflammation by diminishing MMP-10 expression in the lung.

The loss of STAT3 expression greatly impaired innate anti-inflammatory responses in the lung. SOCS3 expression is positively regulated by STAT3 and, once activated, SOCS3 binds gp130 and inhibits Janus activated kinase (JAK) activity. Through this mechanism, SOCS3 then modulates immune responses by down regulating IL-1 and IL-6 signaling (Croker et al., 2003; Frobose et al., 2006) and deterring MAPK activation (Puhr et al., 2010). Thus, SOCS blocks key factors in the pathogenesis of COPD (Mercer et al., 2004; Botelho et al., 2011). The loss of SOCS3 expression in macrophages causes a polarization into an M1 or pro inflammatory phenotype (Qin et al., 2012a) and can enhance cell death and apoptosis (Ruan et al., 2010; Qin et al., 2012b). This may explain the increased macrophage levels and heightened apoptotic responses observed in our study. Aside from SOCS3, basal levels of the anti-inflammatory cytokine IL-10 were significantly lower in the STAT3^−/−^ mice. IL-10 is positively regulated by STAT3 (Benkhart et al., 2000) and IL-10 deficiency renders the lung more sensitive to pro inflammatory stimuli (Penttila et al., 2008). Thus, we assert that the loss of STAT3 expression enhanced susceptibility to smoke-induced inflammation by decreasing IL-10 levels in the lung. Lastly, the STAT3^−/−^ mice did not increase MMP-10 expression in response to cigarette smoke exposure. While most MMPs are believed to promote lung inflammation, MMP-10 exerts anti-inflammatory effects and promotes repair responses following tissue injury (Rodriguez et al., 2008; Koller et al., 2012). Together, these results show that the loss of STAT3 expression causes deficient anti-inflammatory and repair processes in mice.

There are several study limitations that merit discussion. For one, constitutive STAT3^−/−^ mice were utilized. STAT3 plays an important role in myeloid cell maturation and differentiation (Smithgall et al., 2000). Thus, the lack of STAT3 activation may have altered the development of the immune system to render the STAT3^−/−^ mice more responsive to cigarette smoke exposure. Moreover, since a whole body knockout was used the study cannot address the effect of STAT3 loss specifically in the lung. Lastly, though the loss of STAT3 exacerbated inflammation, protease expression and apoptosis, it is uncertain whether these effects would have increased lung tissue destruction in the smoke-exposed mice. Long-term smoke exposure studies and complete morphological analyses are needed to address this question.

In summary, our findings show that STAT3 is activated by cigarette smoke exposure, which may regulate key inflammatory, proteolytic and apoptotic responses in the lung. STAT3 mediates these effects at least in part by modulating anti-inflammatory responses such as SOCS3 and IL-10 expression in the lung. Future studies are needed to address the role of STAT3 in the disease to determine whether targeting STAT3 activity could be used as an approach to counter the injurious effects of cigarette smoke exposure in the lung.

## Author contributions

Patrick Geraghty, Anya Wyman, Itsaso Garcia-Arcos, Abdoulaye J. Dabo and Sonya Gadhvi conducted the animal studies and interpreted experimental results. Patrick Geraghty and Itsaso Garcia-Arcos wrote the paper. Robert Foronjy led the project, interpreted the data and wrote the paper.

### Conflict of interest statement

The authors declare that the research was conducted in the absence of any commercial or financial relationships that could be construed as a potential conflict of interest.
